# Could Periodontitis Increase the Risk of Suffering from Pancreatic Cancer?—A Systematic Review

**DOI:** 10.3390/cancers16071257

**Published:** 2024-03-23

**Authors:** Cecilia Fabiana Márquez-Arrico, Francisco Javier Silvestre, Julia Elena Marquez-Arrico, Javier Silvestre-Rangil

**Affiliations:** 1 Stomatology Department, University of Valencia, 46010 Valencia, Spain; silves@uv.es (F.J.S.); silranja@uv.es (J.S.-R.); 2 Doctor Peset University Hospital, University of Valencia, 46017 Valencia, Spain; 3 Department of Clinical Psychology and Psychobiology, University of Barcelona, 08035 Barcelona, Spain; jmarquez@ub.edu; 4 Institut de Neurociències, University of Barcelona, 08035 Barcelona, Spain

**Keywords:** pancreatic cancer, periodontitis, inflammation, *Porphyromona gingivalis*

## Abstract

**Simple Summary:**

Through this systematic review, we analyzed whether the scientific literature supports the association between pancreatic cancer and periodontitis. We identified three main paths for this association: (1) the presence of common risk factors in both diseases, such as tobacco, obesity, aging, chronic stress and the presence of chronic systemic inflammatory pathologies; (2) changes in the intestinal flora caused by periodontal pathogens, affecting its physiological functions and increasing the risk of cancer; and (3) the cytotoxic effects of periodontitis leading to epigenetic changes, which are linked to the risk of cancer. To summarize, the available literature reviews suggest that both pathologies are linked, and that the presence of periodontitis can worsen the prognosis of pancreatic cancer.

**Abstract:**

(1) Background: The relationship between periodontitis and systemic pathologies continues to grow. Recently, the presence of periodontal pathogens has been linked to an increased risk of pancreatic cancer (PC) and its mortality. Thus, a systematic review is needed to identify whether an association between the two diseases can be established. The objective of this review is to elucidate the mechanisms responsible for this association. (2) Methods: A systematic review was carried out using three databases (PubMed, Embase and Scopus) with the following keywords “Periodontitis AND pancreatic cancer”. A total of 653 articles were retrieved; before selection and screening, the inclusion and exclusion criteria were defined, resulting in a total of 13 articles being included in the review. (3) Results: The increase in low-grade systemic inflammation, pH changes, and the cytotoxicity of certain periodontopathogenic bacteria were found in the scientific literature reviewed as mechanisms linking periodontitis with the risk of PC. (4) Conclusions: Through this systematic review, we have seen how periodontitis can be related to PC and how it worsens its prognosis. Knowing the behavior of periodontopathogenic bacteria and the influence they have on our immune and inflammatory system may help to achieve an interdisciplinary approach to both pathologies.

## 1. Introduction

Periodontitis is a chronic multifactorial inflammatory disease associated with dysbiotic plaque biofilms causing progressive destruction of the supporting tooth tissues [1]. A series of inflammatory events occur, leading to the activation of host-derived proteinases that destroy the fibers of the periodontal ligament, the apical migration of the junctional epithelium, and the formation of periodontal pockets, with a dysbiotic film along the root surface generating more inflammation [2].

Nowadays, periodontitis is a major health problem, reaching a prevalence of up to 90% in older adults [3], and it is favored by previous changes in the oral microbiome [4], producing a dysbiosis that favors inflammation and feeds back [5,6,7]. 

It is known that tissue colonization by periodontopathogenic bacteria can cause systemic health problems [8,9]. Periodontitis can generate an increase in systemic inflammation due to the response that this pathology causes in our immune system [8]. In fact, patients with periodontitis in advanced stages show changes in hemodynamics and leukocyte–endothelial interaction, and are more vulnerable to the development of cardiovascular, metabolic pathology and alterations in the cell cycle [8,9,10]. One of the most studied periodontopathogenic bacteria is *Porphyromona gingivalis* (*P. gingivalis*) [9,10].

*P. gingivalis* is one of the germs involved in the biofilm changes and in the formation of the dysbiotic bacterial plaque [9,10,11]. Although, a part of the interaction with the host and the immune response that is produced depends on the immune response of the susceptible host [9,10,11].

*P. gingivalis* is a Gram-negative, anaerobic coccobacillus that presents structural fimbriae that facilitate its adhesion to tissues [9,10,11,12]. Moreover, it secretes enzymes such as proteases that can cause tissue damage and lipopolysaccharides (LPS) that are associated with systemic diseases [9,10]. *P. gingivalis* presents a capsular polysaccharide that is related to the evasion of the host’s immune system. The capsulated strains have a greater resistance to phagocytosis and generate a lower induction of the alternative pathway of complement [13]. These capsulated strains have a higher resistance to phagocytosis and generate a lower induction of the alternative pathway of complement [13].

Furthermore, pancreatic cancer (PC) is the fourth tumor in mortality and its 5-year survival is below 9% in all its stages [12]. This complicated prognosis is due to several factors, such as the lack of blood biomarkers for early diagnosis or individualized treatment and the observed resistance to chemotherapy in several samples [12,14,15]. As it is known, periodontitis is related to multiple systemic diseases, especially those of a chronic inflammatory type such as diabetes, obesity, metabolic syndrome, and polycystic ovary syndrome [16,17,18,19]. Recently, some research has raised the association of periodontitis with the prevalence of several types of cancer [12,14,15,20,21]. Specifically, many epidemiological studies [15,22] have related the risk of suffering from PC with various factors such as tobacco, overweight, diabetes and, lately, periodontal disease [23].

PC and periodontitis are two significant health concerns with potential interconnections. Among the different theories that link the association between PC and periodontitis, the presence of risk common factors to both diseases stands out, highlighting variables like age [24], diabetes, tobacco use, poor diet, and chronic stress [25]. In addition, it was observed that reactive oxygen species increase inflammatory activity, and bacterial dysbiosis may be present in both diagnoses, generating a reciprocal positive feedback loop between them [1,20,21,22,23,26]. Despite the fact that recent studies have suggested a potential link between PC and periodontitis, the nature and specificity of this association remains unclear. Understanding the relationship between these two conditions could provide valuable insights into their prevention, diagnosis, and management. Therefore, the aim of this systematic review is to synthetize the available published evidence regarding the association of both pathologies and to identify the possible pathways of association between both diseases.

## 2. Materials and Methods

### 2.1. Protocol and Literature Search

**Figure 1:**
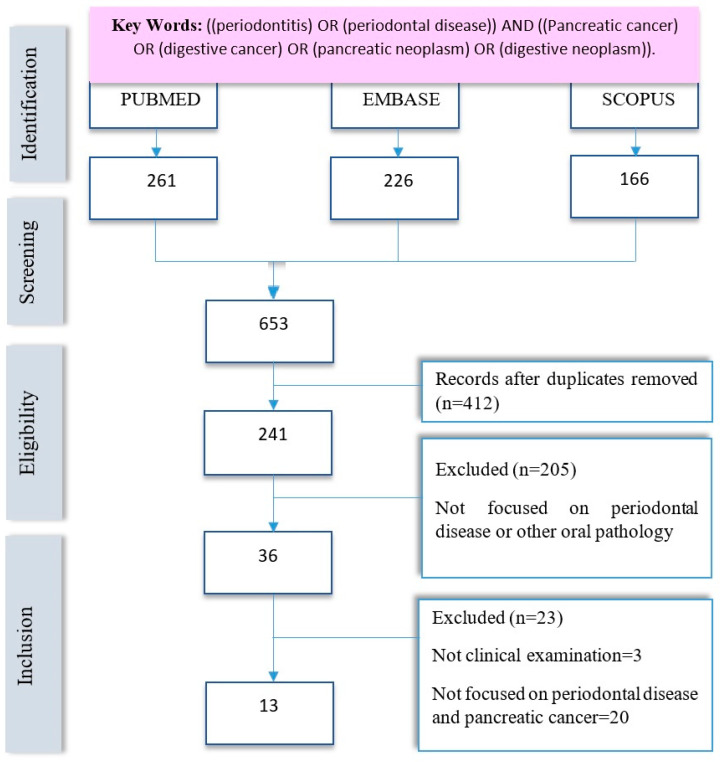
Flow chart following the PRISMA GUIDE.

A systematic review was carried out using the PRISMA guide in its latest version [27]. This revision was registered in the OSF Records with the code osf.io/g23hv and the following direct link to all the information of the revision carried out: URL https://doi.org/10.17605/OSF.IO/ZXDPV (accessed on 29 February 2024). In order to analyze the up-to-now literature about the possible associations between PC and periodontitis, the following search strategy was delivered in PubMed, Embase and Scopus: ((periodontitis) OR (periodontal disease)) AND ((Pancreatic cancer) OR (digestive cancer) OR (pancreatic neoplasm) OR (digestive neoplasm)). A total of 653 publications (articles) were found without applying any filter. After limiting the results to publications from 2011 to the present date, 2023, a total of 241 articles were obtained. Before proceeding to the selection and screening of the articles, the inclusion and exclusion criteria were defined, resulting in a total of 13 articles to be included in this review (Figure 1). The authors developed the PRISMA checklist, which is available in the Supplementary Materials.

### 2.2. Inclusion Criteria

To unify the diagnostic criteria for the PC reviewed in this research, the search focused on malignant epithelial tumors, according to the current WHO classification [28] that includes ductal adenocarcinoma; acinar cell carcinoma; pancreatoblastoma; and solid pseudopapillary neoplasm. We included articles published between 2011 and 2023, considering original research studies such as clinical trials, cohorts, and case–control studies. The sample size was considered, including articles with a sample size ≥30 individuals. A critical reading of each manuscript was carried out, including those that were focused on periodontitis and its relationship with PC.

### 2.3. Exclusion Criteria

Exclusion criteria were as follows: experimental studies carried out on non-human samples; studies focused on other types of cancer in regions of the digestive system other than the pancreas, such as oral cavity, esophagus, stomach, colon or any type of injury or precancerous lesions. Moreover, we excluded articles whose design was out of review or those relating periodontitis with any other type of systemic disease. We also eliminated case reports or series of cases (n < 30).

### 2.4. Data Extraction and Synthesis

Data extraction was performed independently by two reviewers (CFM-A and FJS) using a standardized form. Extracted information included study characteristics (e.g., author, year of publication, study design), participant characteristics (e.g., sample size), exposure and outcome variables (e.g., measures of periodontal disease, pancreatic cancer diagnosis), and key findings. Any discrepancies will be resolved through discussion or consultation with a third reviewer (JEM-A or JS-R).

### 2.5. Quality Assessment

To analyze the selected articles, the quality scales corresponding to their methodological design were passed. In the case of case–control, cohort, and cross-sectional studies, we used the Ottawa scales [29]. For the in vitro studies, we used the QUIN Scale [30]. All the articles showed a high-quality approach, obtaining high marks on their respective scales (Table 1, Table 2, Table 3 and Table 4).

## 3. Results

In total, 13 articles were found associating periodontal disease with an increased prevalence of PC compared to healthy controls without associated periodontal pathology [10,12,14,15,20,23,26,31,32,33,34,35] (Table 5). The reviewed publications include a total of 1728 patients with PC. We analyzed 5 case and control studies [20,23,31,32,33], 3 cohort studies [12,15,34], 2 cross-sectional [22,35] and 3 in vitro studies [10,14,26].

In general, all the articles find a significative relationship between both pathologies. In the reviewed literature, we identified three main paths for this association: (1) the presence of common risk factors in both diseases, such as tobacco, obesity, aging, chronic stress and the presence of chronic systemic inflammatory pathologies [22,23]; (2) studies indicating how the periodontal pathogens can cause changes in the intestinal flora, affecting its physiological functions, increasing the risk of cancer [10,14,22,26,31,34,35] and; (3) the cytotoxic effects of periodontitis leading to epigenetic changes linked to the risk of cancer [12,14,23,26,35].

Among the mechanisms linking periodontitis with the increased risk of PC, we find that the consequences generated by the increase in periodontopathogenic bacteria seem to be a key factor. This bacterial dysbiosis produces cellular changes in response to increased oxidative stress, changes in pH, and the cancellation of inhibitory mechanisms of oncogenesis. All of this implies creating an environment that favors cellular changes to the detriment of anticarcinogenic protective factors. *P. gingivalis* modifies the inflammatory tumor microenvironment and recruits neutrophils and enhances neutrophil elastase secretion to promote PC [20,31]. Everything points to the fact that alterations in the oral flora towards a more aggressive one, with a predominance of anaerobic Gram-negative periodontopathogens, may facilitate the colonization of said pathogens in other areas of the body, such as the gastro-digestive area [10,14,26]. The infection of these bacteria in pancreatic cells can alter the natural cycle of the cells, increasing their inflammation and cancerous processes [23,26,31,34].

The article by Miskiewiez et al., 2018 [23], shows how periodontitis in patients with PC was independent of the oral hygiene status. That is to say, even in those patients who presented low plaque indices, periodontal involvement was found; enzyme activity (lipase and amylase) and chronic pancreatitis were interrelated [23,32]. In addition, in cross-sectional studies, we see that patients with PC and periodontitis have increased mortality rates [13,35]. This fact could be attributed to an exacerbated response of their immune system due to the low-grade systemic inflammation present in patients with advanced periodontitis.

Therefore, bacterial dysbiosis would affect generating changes at three levels: at the extracellular level (changes in pH lead to an increase in free radicals due to increased oxidative stress, as well as an increase in TNFα and interleukins, among others); in signaling pathways (notably, the inhibition of p53); and in DNA (such as the promotion of tumor development and mutations). It is noteworthy that only one study [15] found no association between severe periodontitis and PC.

Proteolytic enzymes have recently acquired great interest in tumor pathophysiology due to their potential role in the degradation of the main components of the extracellular matrix and basement membrane, facilitating tumor invasion and metastasis. In their study, Nieminen et al., 2018 [14], presented how the receptor of the periodontopathogenic bacteria Treponema denticula (Td-CTLP) was able to degrade the proteinase inhibitors TIMP-1, TIMP-2 and α-1-antichymotrypsin. Hence, the data showed how the bacterial infection, in addition to making changes in the patient’s hemodynamics, generates an inhibition of the protective systems of the cell cycle.

## 4. Discussion

Through this systematic review of publications about the possible associations between PC and periodontitis, some relationships were found to be relevant in the available literature regarding periodontal germs such as *P. gingivalis*, *Tannerella forsythia*, and *Fusobacterium nucleatum* with oral dysbacteriosis and various types of digestive cancer [9,10,35]. The increase in new technologies, such as massive sequencing for the investigation of bacterial groups, has favored the study of the associations between oral dysbiosis and carcinogenesis. However, there are still many gaps regarding the pathogenic pathways and mechanisms that trigger these cellular changes. This is due, in part, to the different characteristics and behavior of the tumors and the heterogeneity of the methodology used.

Moreover, the existence of periodontal bacteria has been observed in certain digestive tumors [9,10,11] proving a certain degree of relationship between PC and periodontitis. One of the most studied bacteria has been *P. gingivalis*, and it is speculated that it can cause some carcinogenic effects related to an altered immune response [4,36,37].

There is a close relationship between the oral microbiome and the microbiome of the digestive tract. In fact, poor oral hygiene has been associated with an increased risk of developing PC [11,34,38], and even a high presence of antibodies against *P. gingivalis* can triple this risk [10,26]. *P. gingivalis* can initiate the Toll-like receptor (TLR) signaling pathway and its complete activation could contribute to the development of pancreatic carcinogenesis, as observed in experimental animal studies [12,26].

Likewise, a high percentage of mutations in the p53 suppressor gene has been observed in patients with PC, and it seems that *P. gingivalis* inhibits epithelial cell apoptosis by increasing p53 activation in the event of germ invasion [20]. Thus, mutations in p53 could play a key role in the relationship between the microorganism and the development of PC [23]. Moreover, the dysbiotic oral microbiota could favor carcinogenesis through the alteration of the extracellular medium by certain germs due to the inflammatory changes and the permanence of free radicals, or due to the acids produced in the cellular microenvironment [13]. Likewise, the dysbiotic oral microbiota can contribute to the carcinogenesis process by the activation of second messenger pathways and by the affectation of the DNA acting directly or by associated proteins generating epigenetic changes [4,11,26].

It is crucial to mention that patients with periodontitis often present local and systemic risk factors that may be common to the increased risk of cancer. Tobacco, alcohol, drug use, nutrition low in antioxidants, a sedentary lifestyle and other lifestyle factors can be linked to both pathologies [39,40]. However, in the studies analyzed, oral hygiene indices and smoking were considered as confounding variables, and the presence of other systemic pathologies was an exclusion criterion.

In oral squamous cell cancer, it has been observed that the presence of *P. gingivalis* accelerates the cell cycle and suppresses apoptosis, although macroautophagy is increased [1,23,33]. Therefore, *P. gingivalis* could facilitate the proliferation of tumor cells by affecting defensin expression genes through peptidyl arginine deiminase and the activation of β-catenin. Also, it can convert ethanol to acetaldehyde, leading to the consideration of a direct relationship between *P. gingivalis* and digestive cancer, even in the absence of periodontal disease. These bacteria present general mechanisms involved in the cellular metabolism related to carcinogenesis, such as a decrease in pH, an increase in free radicals and an increase in TNFα [41,42,43,44,45]. Furthermore, the presence of periodontitis is related to an increase in mortality rates [46,47]. 

On the other hand, cellular toxicity can occur due to the increase in *P. gingivalis* colonies that determine the inhibition of tumor suppressor genes such as p53 [12,14,26]. Likewise, the inhibition of apoptosis in epithelial cells could enhance the immune evasion of tumor cells and the following production of carcinogenic substances [10,12,23]. In experimental studies, it has been observed that the implementation of periodontal pathogenic bacteria causes significant changes in the intestinal flora with a dysbiosis that can affect intestinal functions [10,15]. Recent reviews have supported this association between cellular cytotoxicity produced by periodontitis and an increased risk of pancreatic cancer [46,47,48]. 

In addition to cellular toxicity, direct epigenetic damage to epithelial cells can occur. Mitochondrial metabolism is altered, leading to an increase in free radicals and suppression of some antitumor mechanisms, which is why, in addition to pancreatic cancer being linked to periodontitis, other cancers such as breast, lung, and prostate have been associated with periodontitis. Certain phenotypic changes have been demonstrated in mononuclear cells, leading to the release of said free radicals and cytokines, as well as the degradation of the extracellular matrix, all mechanisms involved in carcinogenic and metastatic processes [46,47,48].

Furthermore, it is currently important to know the composition and ecology of the PC microbiota, where an exchange of bacteria between saliva and PC has recently been proven with a possible migration of *P. gingivalis* from the oral microbiome to the digestive tract and to other areas of the body through vascularization [46,47,48]. The latter has been demonstrated in experimental studies with 16s rRNA [10,49,50] sequencing and highlights the relevance of the presence of periodontopathogenic bacteria in digestive tumors.

In a previous study, it was possible to verify microbial communities at various taxonomic levels between both locations [20]. Various routes for such microbial colonization to the pancreas via the gastrointestinal tract or via the mesenteric lymph nodes have been proposed [20]. 

This colonization could promote carcinogenesis in the pancreatic tissue and, therefore, increase the risk of having PC. In this sense, the oncogenic potential of *P. gingivalis* has recently begun to be elucidated. The promotion of tumorigenesis has been observed in murine models, which is why Gnanasekaran et al. [26] demonstrated that *P. gingivalis* promoted the proliferation of early-stage PC cell lines, improving under hypoxic conditions. Research has demonstrated that *P. gingivalis* can employ several virulence factors favoring an escape from the immune system, affecting not only cell-signaling mechanisms, but also indirectly dampening host immunity and altering cytokine production [37].

After the invasion of *P. gingivalis* in the tumor, there is a significant increase in neutrophils and a decrease in T cells (CD8+) [25]. There is a strong release of chemokines from neutrophils that could be related to tumor initiation and progression [51]. NET-associated proteases, including neutrophil elastase and MPO, have been shown to be significantly increased after *P. gingivalis* infection, although these mechanisms remain unclear [52,53].

## 5. Conclusions

Through this systematic review, we have seen how periodontitis can be related to pancreatic cancer, worsening its prognosis. It is important to know the behavior of periodontopathogenic bacteria and the influence they have on our immune and inflammatory system to achieve an interdisciplinary approach to both pathologies.

## Figures and Tables

**Table 1:** Quality analysis of the studies using the Newcastle–Ottawa Scale for case–control studies [29].

Authors	Selection	Comparability	Exposure	Total Score/9
Fan et al., 2017 [31]	*	*	**	4/9
Miskiewicz et al., 2018 [23]	***	**	**	8/9
Miskiewicz et al., 2015 [32]	***	**	**	7/9
Tan et al., 2022 [20]	**	*	*	6/9
Vogtmann et al., 2019 [33]	***	**	**	7/9

Selection: the score in this section depends on the representativeness of the exposed cohort, ascertainment of exposure and demonstration that the outcome of interest was not present at start of study. Comparability: comparability of cases and controls on the basis of the design or analysis. Exposure: ascertainment of exposure, with objective methods, such as surgical procedures or structured interviews, where the assessor remains blind to the case/control status. Same method of ascertainment of cases and controls. Same response rate to treatment or procedure for both groups. *: 1 point; ** 2 points, *** 3 points of total score.

**Table 2:** Quality analysis of the studies using the Newcastle–Ottawa Scale for cohort studies [29].

Authors	Selection Items				Comparability Items	Exposure Items			Total Score/9
	1	2	3	4	5	6	7	8	
Chou S-H et al., 2018 [15]	*	*	*		*	*	*	*	7/9
Heikkilä P et al., 2018 [12]	*	*	*		**	*	*		7/9
Michaud et al., 2013 [34]	*	*	*	*	*	*			7/9

Selection: representativeness of the exposed cohort *, selection of the non-exposed cohort *, ascertainment of exposure *, demonstration that the outcome of interest was not present at the start of the study *; comparability: comparability of the ability of cohorts on the basis of the design or analysis **, and outcome: assessment of outcome *, with follow-up over a duration long enough for outcomes to occur *, adequacy of the follow-up of cohorts *. * 1 point, ** 2 points of total score.

**Table 3:** Quality analysis of the studies using the Newcastle–Ottawa Scale for cross-sectional studies [29].

Authors	Selection Items				Comparability Items	Outcomes Items		Total Score/9
	1	2	3	4	5	6	7	
Gerlovin et al., 2019 [22]	*	*	*			*	**	6/9
Kei et al., 2015 [35]	*	*	*			*	**	6/9

Selection: 1: Representativeness of the sample (1 point); 2: Sample size (1 point); 3: Non-respondents (1 point); 4: Ascertainment of exposure (2 points); 5: Comparability: subjects in different outcomes are comparable, based on the study design or analysis, and confounding factors are controlled (1 point); 6: Outcome: Assessment of the outcome (2 points); 7: Statistical test (1 point). *: Corresponds to 1 point from the total score when the research adequately meets the item. * 1 point; ** 2 points of the total score.

**Table 4:** Quality analysis of the studies using the QUIN Scale for cross in vitro studies [30].

The QUIN Scale	Authors		
Criteria:	Gnanasekaran et al., 2020 [26]	Nieminen et al., 2018 [14]	Sugiyama et al., 2022 [10]
Clearly stated aims/objectives	2	2	2
Detailed explanation of sample size calculation	0	0	1
Detailed explanation of sampling technique	2	2	1
Details of comparison group	N/A	N/A	N/A
Detailed explanation of methodology	2	2	1
Operator details	1	2	1
Randomization	0	0	0
Method of measurement of outcome	2	2	2
Outcome assessor details	2	2	2
Blinding	0	0	0
Statistical analysis	2	2	2
Presentation of results	2	2	2

Adequately specified (Score = 2); Inadequately specified (Score = 1); Not specified (Score = 0); Not applicable (N/A).

**Table 5:** General characteristics of the studies analyzed.

Author, Year	Study Design	Sample Size	Principal Findings
Miskiewicz et al., 2015 [32]	Case control study	Pancreatic cancer n = 18, chronic pancreatitis n = 39, controls n = 119	The activation of the NLRP3 inflammasome present in subjects with periodontitis and pancreatic cancer is analyzed. This activation is linked to both pathologies. A genomic study of this receptor is carried out. All periodontal parameters (BOP and CAL) were significantly worse (*p* = 0.001 and *p* = 0.001, respectively) in patients with chronic pancreatitis than in the two other groups. The NLRP2 polymorphism was associated with chronic pancreatitis, whereas the NLRP3 polymorphism was comorbid with pancreatic cancer and the increase of CAL.
Chou et al., 2018 [15]	Retrospective Cohorts	n = 25,485 individuals with periodontitis. Gastrointestinal cancers: 275 mild periodontitis; 324 severe periodontitis	Severe periodontitis not associated with an increased risk of total individual gastrointestinal cancers compared con mild periodontitis
Fan et al., 2017 [31]	Case control study	Pancreatic cancer cases= 361 Control = 371 (were drawn from 2 cohorts the American Cancer Society and National Cancer Institute Prostate, Lung, Colorectal and Ovarian Cancer Screening Trial)	*P. gingivalis* and Aa > pancreatic cancer risk (OR 1.60). *Phylum Fusobacteria* and genus *Leptotrichia* < risk. Oral microbiota may be a role pancreatic cancer aetiology.
Gerlovin et al., 2019 [22]	Cross sectional study (with a Biennial follow-up questionnaire by mail).	n = 59,000 African American women	A total of 78 incidents of pancreatic cancer occurred during follow-ups from 2007 through 2016, with participants contributing an average of 9.85 years of follow-up. Relative to the reference category of women who never reported either tooth loss or periodontal disease, multivariable HRs were 1.77 for periodontal disease with no tooth loss, 1.58, for periodontal disease with tooth loss, and 2.05 for tooth loss without periodontal disease. Results from this study suggest that poor oral health may play a role in racial disparities in pancreatic cancer incidence.
Gnanasekaran et al., 2020 [26]	Experimental study	3 cellular lines of Pancreatic ductal adenocarcinoma (2 humans and 1 mouse cells line). Cells with absence of *P. gingivalis* vs. cells with *P. gingivalis*.	This study focuses on the relationship between the mechanism of cell cycle impairment in the event of superinfection by periodontopathogens. Specifically, this study is the first to test the mechanistic involvement of *P. gingivalis* in pancreatic tumorigenesis, applying in vitro tools and a xenograft pancreatic carcinoma model in vivo. Our results reveal a previously unknown direct effect of *P. gingivalis* on PDAC progression, highlighting the importance of the interplay between hypoxia and *P. gingivalis* intracellular survival. *P. gingivalis* infection enhances PDAC cell proliferation.
Heikkila et al., 2018 [12]	Cohort Longitudinal study	n = 68,273 Periodontal status was defined based on periodontal treatment procedure codes.	This research analyzes the reported history of dental status (number of teeth, health indices, initial caries, decayed/missing/filled teeth) and CPITN. Furthermore, it defines periodontitis as a binary variable (no/yes) based on the above codes. Data support the association between periodontitis and mortality in all types of cancer, especially pancreatic cancer.
Michaud et al., 2013 [34]	Cohort study to select cases and control from a data base	Blood samples from 405 pancreatic cancer cases and 416 matched controls.	This study performed blood tests to detect antibodies to periodontal bacteria. Individuals with high levels of antibodies against *P. gingivalis P. gingivalis* ATTC 53,978, a pathogenic periodontal bacterium, had a 2-fold higher risk of pancreatic cancer than individuals with lower levels of these antibodies (odds ratio [OR], 2.14; 95% confidence interval [CI], 1.05–4.36; >200 ng/mL vs. ≤200 ng/mL). People with high levels of antibodies against common oral bacteria had a 45% lower risk of pancreatic cancer compared to those with a profile of lower antibody levels. Periodontal disease might increase the risk of pancreatic cancer.
Miskiewiez et al., 2018 [23]	Case control study	Evaluated oral health level and periodontal status among 3 groups: n = 29 cancer pancreas; n = 41 chronic pancreatitis; n = 50 controls	Both pathologies are linked based on the systemic inflammatory mediators present in the blood and the correlation with local inflammation measured with the BOP. Periodontitis in pancreatic cancer is independent of the state of oral hygiene. BOP 62.5%, enzyme activity (lipase and amilasa) and chronic pancreatitis were interrelated.
Kei et al., 2015 [35]	Transversal study	283 pancreatic cancer tissue specimens	The presence of periodontopathogens is detected as biomarkers of malignancy in pancreatic tumors. This study associates bacterial tissue infection with cell cycle changes and carcinogenic potential. Periodontal bacteria were found in those samples from patients with the highest risk. The presence of FN was in 25 samples (8.8%) and largely coincided with high mortality rates.
Nieminen et al., 2018 [14]	Experimental study	149 orodigestive tumor tissue samples Tissue samples comprised squamous cell carcinomas (SCCs) of tongue (n = 29), tonsil (n = 25), esophagus (n = 3), and adenocarcinoma of stomach (n = 32), pancreas (n = 6), and colon (n = 54).	This study associates bacterial tissue infection with cell cycle changes and the inhibition of protective factors. Td-CTLP was present in the majority of orodigestive tumor samples. Td-CTLP was found to convert pro MMP-8 and -9 into their active forms. In addition, Td-CTLP was able to degrade the proteinase inhibitors TIMP-1, TIMP-2, and a-1-antichymotrypsin, as well as complement C1q.
Sugiyama et al., 2022 [10]	Experimental study	2 groups of 5 mice: Received *P. gingivalis P. gingivalis* -LPS and controls. Effect on *P. gingivalis* -LPS on the gut flora	Infection with periodontopathogens increases TFNα and other inflammatory mediators that are also increased in cancer patients. This route of infection increases the risk of developing cancerous pathologies. The administration of periodontal pathogens can cause changes in the intestinal flora, affecting its physiological functions increasing the risk of cancer.
Tan et al., 2022 [20]	Case control study	Study intrapancreatic microbiome composition on resected cancer tissue and matched normal adjacent tissues	In this research, a path of association is indicated due to the increase in low-grade systemic inflammation that occurs due to periodontitis because of the invasion of *P. gingivalis*. Neutrophils (main cells of the innate response to bacterial aggression) are secreters of elastase and are involved in cellular changes that occur in cancer. *P. gingivalis* modifies the inflammatory tumor microenvironment and recruits neutrophils and enhances the secretion of neutrophils elastase to promote cancer pancreatic
Vogtmann et al., 2019 [33]	Case control study	A total of 273 pancreatic adenocarcinoma cases and 285 controls	The abundance of some specific microbial taxa was also associated with pancreatic cancer, including *Haemophilus, Enterobacteriaceae, Lachnospiraceae* G7, *Bacteroidaceae*, and *Staphylococcaceae*. The microbial community and taxa level differences could be related to the presence of pancreatic cancer or the risk of developing pancreatic cancer. *P. gingivalis* was not associated with pancreatic cancer and was detected in 76.92% of cases and 76.49% of controls.

Aa: *Aggregatibacter actinomycetemcomitans*; BOP: bleeding on probing; CAL: clinical attachment loss; CPITN: Community Periodontal Index of Treatment Needs from WHO; FN: *Fusobacterium nucleatum*; MMP: matrix metalloproteinases; NLRP: nod-like receptor pyrin domain; PDAC: pancreatic ductal adenocarcinoma; *P. gingivalis*: *Porphyromonas gingivalis*; Td-CTLP: *Treponema denticola* chymotrypsin-like proteinase; TIMP: tissue inhibitors of matrix metalloproteinases.

## Data Availability

The data presented in this study are available in this article and Supplementary Materials.

## Supplementary Materials

The following supporting information can be downloaded at: https://www.mdpi.com/article/10.3390/cancers16071257/s1, Table S1: PRISMA 2020 Checklist [54].
