# Supplementary material for: Could Periodontitis Increase the Risk of Suffering from Pancreatic Cancer?—A Systematic Review
Source: Cancers (Basel). 2024 Mar 23;16(7):1257. doi: 10.3390/cancers16071257 (PMC11010905; doi:10.3390/cancers16071257)
Supplement: Supplementary file 1 [file cancers-16-01257-s001.zip › cancers-2900476-supplementary.pdf]

Table S1. PRISMA 2020 Checklist.

Section and Topic	Item#	Checklist item	Location where item is reported
**TITLE**			
Title	1	Identify the report as a systematic review.	Page 1.
**ABSTRACT**			
Abstract	2	See the PRISMA 2020 for Abstracts checklist.	Page 1.
**INTRODUCTION**			
Rationale	3	Describe the rationale for the review in the context of existing knowledge.	Page 2.
Objectives	4	Provide an explicit statement of the objective(s) or question(s) the review addresses.	Page 2.
**METHODS**			
Eligibility criteria	5	Specify the inclusion and exclusion criteria for the review and how studies were grouped for the syntheses.	Page 3.
Information sources	6	Specify all databases, registers, websites, organizations, reference lists and other sources searched or consulted to identify studies. Specify thedate when each source was last searched or consulted.	Page 2.
Search strategy	7	Present the full search strategies for all databases, registers and websites, including any filters and limits used.	Pages 2 and 3.
Selection process	8	Specify the methods used to decide whether a study met the inclusion criteria of the review, including how many reviewers screened each recordand each report retrieved, whether they worked independently, and if applicable, details of automation tools used in the process.	Page 3.
Data collection process	9	Specify the methods used to collect data from reports, including how many reviewers collected data from each report, whether they worked independently, any processes for obtaining or confirming data from study investigators, and if applicable, details of automation tools used in the process.	Page 3.
Data items	10a	List and define all outcomes for which data were sought. Specify whether all results that were compatible with each outcome domain in eachstudy were sought (e.g. for all measures, time points, analyses), and if not, the methods used to decide which results to collect.	Pages 3 and 5.
	10b	List and define all other variables for which data were sought (e.g. participant and intervention characteristics, funding sources). Describe any assumptions made about any missing or unclear information.	Pages 3 and 5.
Study risk of bias assessment	11	Specify the methods used to assess risk of bias in the included studies, including details of the tool(s) used, how many reviewers assessed each study and whether they worked independently, and if applicable, details of automation tools used in the process.	Page 4.
Effect measures	12	Specify for each outcome the effect measure(s) (e.g. risk ratio, mean difference) used in the synthesis or presentation of results.	Page 5.
Synthesis methods	13a	Describe the processes used to decide which studies were eligible for each synthesis (e.g. tabulating the study intervention characteristics andcomparing against the planned groups for each synthesis (item #5)).	Pag 4.
	13b	Describe any methods required to prepare the data for presentation or synthesis, such as handling of missing summary statistics, or dataconversions.	Page 4.
	13c	Describe any methods used to tabulate or visually display results of individual studies and syntheses.	Page 5.
	13d	Describe any methods used to synthesize results and provide a rationale for the choice(s). If meta-analysis was performed, describe themodel(s), method(s) to identify the presence and extent of	Pages 6, 7 and 8.

		statistical heterogeneity, and software package(s) used.	
	13e	Describe any methods used to explore possible causes of heterogeneity among study results (e.g. subgroup analysis, meta-regression).	N/A
	13f	Describe any sensitivity analyses conducted to assess robustness of the synthesized results.	N/A
Reporting bias assessment	14	Describe any methods used to assess risk of bias due to missing results in a synthesis (arising from reporting biases).	N/A
Certainty assessment	15	Describe any methods used to assess certainty (or confidence) in the body of evidence for an outcome.	N/A

From: Page MJ, McKenzie JE, Bossuyt PM, Boutron I, Hoffmann TC, Mulrow CD, et al. The PRISMA 2020 statement: an updated guideline for reporting systematic reviews. *BMJ* **2021**; 372: n71. https://doi: 10.1136/bmj.n71
